# 754 The Effect of the COVID-19 Pandemic on Burn Care and Short-Term Patient Outcomes

**DOI:** 10.1093/jbcr/irac012.307

**Published:** 2022-03-23

**Authors:** Jacquelyn Withers, Jodi Gedallovich, Richard Grossman

**Affiliations:** University of California San Francisco, San Francisco, California; University of California San Francisco, San Francisco, California; Saint Francis Memorial Hospital, Bothin Burn Center, San Francisco, California

## Abstract

**Introduction:**

The COVID-19 pandemic has forced profound changes on many aspects of American healthcare delivery. Resource utilization and risk minimization have been the primary goals behind these shifts, and adaptations made to optimize public safety continue to affect patients. It is not known, however, how these changes have impacted burn patients. The aim of this study is to detect any effects the pandemic has had on this population by describing the incidence, nature, and short-term outcomes of patients treated by a single surgeon at a major burn center during the area’s shelter-in-place period.

**Methods:**

A retrospective cohort study was performed using a database of one surgeon’s (RG) admissions and surgical procedures. All patients treated for acute burn injuries within the year following the announcement of COVID-associated shelter-in-place orders in the burn center’s area (March 2020-March 2021) were included. The control group consisted of the same surgeon’s patients treated in the prior year (March 2019-March 2020). All patients were included regardless of age. Patients treated for other conditions such as dermatologic issues or chronic burn sequelae were excluded. Delayed presentation was defined as an interval longer than 24 hours between injury and first medical encounter. Descriptive analyses were performed to compare the demographics, timing of presentation, treatment courses, and short-term outcomes between pre-pandemic and COVID period groups.

**Results:**

408 patients were included overall, with 227 admitted pre-COVID and 181 during the pandemic. The only significant difference in demographics between groups was a higher incidence of homelessness in the COVID group (7 vs 13%, p < 0.04). Delayed presentation was not significantly different between groups (15 vs 17%, p=0.75). We found no significant differences between groups in rates of cellulitis or sepsis at presentation (9 vs 10%, p=0.8; 5 vs 8%, p=0.32) or during admission (16 vs 18%, p=0.54; 5 vs 8%, p=0.32). The mean number of surgeries per patient was 2 in both groups. Rates of autografting (62 vs 56%, p=0.24), lengths of stay (16 vs 17 days, p=0.34), readmissions (2 vs 4%, p=0.11), and deaths (2 vs 2%, p=0.74) were also similar. There were several complicated cases of delayed care in the COVID group after burns were evaluated initially via telemedicine, including one patient who presented in septic shock, though this finding did not reach statistical significance.

**Conclusions:**

Our results demonstrate that the pandemic did not have a significant impact on many key aspects of acute burn care in this cohort. Patients in the pandemic period did not delay treatment at a higher rate, and short-term outcomes were comparable overall between groups. Further studies will be useful in understanding the effect of the pandemic and telemedicine on burn care in a broader context.

